# Efficient Proximal Gradient Algorithms for Joint Graphical Lasso

**DOI:** 10.3390/e23121623

**Published:** 2021-12-02

**Authors:** Jie Chen, Ryosuke Shimmura, Joe Suzuki

**Affiliations:** Graduate School of Engineering Science, Osaka University, Osaka 560-0043, Japan; shimmura@sigmath.es.osaka-u.ac.jp (R.S.); j-suzuki@sigmath.es.osaka-u.ac.jp (J.S.)

**Keywords:** Gaussian graphical model, joint graphical lasso, proximal gradient descent method

## Abstract

We consider learning as an undirected graphical model from sparse data. While several efficient algorithms have been proposed for graphical lasso (GL), the alternating direction method of multipliers (ADMM) is the main approach taken concerning joint graphical lasso (JGL). We propose proximal gradient procedures with and without a backtracking option for the JGL. These procedures are first-order methods and relatively simple, and the subproblems are solved efficiently in closed form. We further show the boundedness for the solution of the JGL problem and the iterates in the algorithms. The numerical results indicate that the proposed algorithms can achieve high accuracy and precision, and their efficiency is competitive with state-of-the-art algorithms.

## 1. Introduction

Graphical models are widely used to describe the relationships among interacting objects [1]. Such models have been extensively used in various domains, such as bioinformatics, text mining, and social networks. The graph provides a visual way to understand the joint distribution of an entire set of variables.

In this paper, we consider learning Gaussian graphical models that are expressed by undirected graphs, which represent the relationship among continuous variables that follow a joint Gaussian distribution. In an undirected graph, G=(V,E), and edge set *E* represents the conditional dependencies among the variables in vertex set *V*.

Let $X_{1},\ldots ,X_{p}$ (p≥1) be Gaussian variables with covariance matrix $\Sigma \in R^{p\times p}$, and $\Theta :=\Sigma^{-1}$ be the precision matrix, if it exists. We remove the edges so that the variables $X_{i}$, $X_{j}$ are conditionally independent given the other variables if and only if the (i,j)-th element $\theta_{i,j}$ in Θ is 0:

$$\left\{i,j\right\}\notin E\;\;⟺\;\theta_{i,j}=0\;\;⟺X_{i}⊥\;\;\;⊥X_{j}|X_{V\backslash \{i,j\}},$$

where each edge is expressed as a set of two elements in {1,…,p}. In this sense, constructing a Gaussian graphical model is equivalent to estimating a precision matrix.

Suppose that we estimate the undirected graph from data consisting of *n* tuples of *p* variables and that dimension *p* is much higher than sample size *n*. For example, if we have expression data of p=20,000 genes for n=100 case/control patients, how can we construct a gene regulatory network structure from the data? It is almost impossible to estimate the locations of the nonzero elements in Θ by obtaining the inverse of sample covariance matrix $S\in R^{p\times p}$, which is the unbiased estimator of Σ. In fact, if p>n, then no inverse $S^{-1}$ exists because the rank of $S\in R^{p\times p}$ is, at most, *n*.

In order to address this situation, two directions are suggested:

1. Sequentially find the variables on which each variable depends via regression so that the quasilikelihood is maximized [2].
2. Find the locations in Θ, the values of which are zeros, so that the $\ell_{1}$ regularized log-likelihood is maximized [3,4,5,6].

We follow the second approach because we assume Gaussian variables, also known as graphical lasso (GL). The  $\ell_{1}$ regularized log-likelihood is defined by:

(1) $$\begin{array}{c} \underset{\Theta }{\mathrm{maximize}}\left\{\log\det\Theta -\mathrm{trace}\left(S\Theta \right)-{\lambda ||\Theta ||}_{1}\right\}, \end{array}$$

where tuning parameter λ controls the amount of sparsity, and  ${\left||\Theta |\right|}_{1}$ denotes the sum of the absolute value of the off-diagonal elements in Θ. Several optimization techniques [4,7,8,9,10,11,12] have been studied for the optimization problem of (1).

In particular, we consider a generalized version of the abovementioned GL. For example, suppose that the gene regulatory networks of thirty case and seventy control patients are different. One might construct a gene regulatory network separately for each of the two categories. However, estimating each on its own does not provide an advantage if a common structure is shared. Instead, we use 100 samples to construct two networks simultaneously. Intuitively speaking, using both types of data improves the reliability of the estimation by increasing the sample size for the genes that show similar values between case and control patients, while using only one type of data leads to a more accurate estimate for genes that show significantly different values. Ref. [13] proposed a joint graphical lasso (JGL) model by including an additional convex penalty (fused or group lasso penalty) to the graphical lasso objective function for *K* classes. For example, *K* is equal to two for the case/control patients in the example. JGL includes fused graphical lasso with fused lasso penalty, which encourages sparsity and the similarity of the value of edges across *K* classes, and group graphical lasso with group lasso penalty, which promotes similar sparsity structure across *K* graphs. Although there are several approaches to handling the multiple graphical models, such as those of [14,15,16,17], the JGL is considered the most promising.

The main topic of this paper is efficiency improvement in terms of solving the JGL problem. For the GL, relatively efficient solving procedures exist. If we differentiate the $\ell_{1}$ regularized log-likelihood (1) by Θ, then we have an equation to solve [4]. Moreover, several improvements have been considered for the GL, such as proximal Newton [12] and proximal gradient [10] procedures. However, for the JGL, even if we derive such an equation, we have no efficient way of handling it.

Instead, the alternating direction method of multipliers (ADMM) [18], which is a procedure for solving convex optimization problems for general purposes, has been the main approach taken [13,19,20,21]. However, ADMM does not scale well concerning the feature dimension *p* and number of classes *K*. It usually takes time to converge to a high-accuracy solution [22].

For efficient procedures to solve the JGL problem, ref. [23] proposed a method based on the proximal Newton method only when the penalty term is expressed by fused lasso. The existing method requires expensive computations for the Hessian matrix and Newton directions, which means that it would be expensive to use for high-dimensional problems.

In this paper, we propose efficient proximal-gradient-based algorithms to solve the JGL problem by extending the procedure in [10] and employing the step-size selection strategy proposed in [24]. Moreover, we provide the theoretical analysis of both methods for the JGL problem.

In our proximal gradient methods for the JGL problem, the proximal operator in each iteration is quite simple, which eases the implementation process and requires very little computation and memory at each step. Simulation experiments are used to justify our proposed methods over the existing ones.

Our main contributions are as follows:

- We propose efficient algorithms based on the proximal gradient method to solve the JGL problem. The algorithms are first-order methods and quite simple, and the subproblems can be solved efficiently with a closed-form solution. The numerical results indicate that the methods can achieve high accuracy and precision, and the computational time is competitive with state-of-art algorithms.
- We provide the boundedness for the solution to the JGL problem and the iterates in algorithms, which is related to the convergence rate of the algorithms. With the boundedness, we can guarantee that our proposed method converges linearly.

Table 1 summarizes the relationship between the proposed and existing methods.

The remaining parts of this paper are as follows. In Section 2, we first provide the background of our proposed methods and introduce the joint graphical lasso problem. In Section 3, we illustrate the detailed content of the proposed algorithms and provide a theoretical analysis. In Section 4, we report some numerical results of the proposed approaches, including comparisons with efficient methods and performance evaluations. Finally, we draw some conclusions in Section 5.

Notation: In this paper, ${\left||x|\right|}_{p}$ denotes the $\ell_{p}$ norm of a vector $x\in R^{d}$, ${\left||x|\right|}_{p}:=(\sum_{i=1}^{d}|x_{i}{{|}^{p})}^{\frac{1}{p}}$ for p∈[1,∞), and ${\left||x|\right|}_{\infty }:=\max_{i}\left|x_{i}\right|$. For a matrix $X\in R^{p\times q}$, ${\left||X|\right|}_{F}$ denotes the Frobenius norm, ${\left||X|\right|}_{2}$ denotes the spectral norm, ${\left||X|\right|}_{\infty }:=\max_{i,j}\left|x_{i,j}\right|$, and ${\left||X|\right|}_{1}:=\sum_{i=1}^{p}\sum_{j=1}^{q}\left|x_{i,j}\right|$ if not specified. The inner product is defined by $\langle X,X\rangle :=\mathrm{trace}\left(X^{T}X\right)$.

## 2. Preliminaries

This section first reviews the graphical lasso (GL) problem and introduces the graphical iterative shrinkage-thresholding algorithm (G-ISTA) [10] to solve it. Then, we introduce the step-size selection strategy that we apply to the joint graphical lasso (JGL) in Section 3.2.

### 2.1. Graphical Lasso

Let $x_{1},\ldots ,x_{n}\in R^{p}$ be n≥1 observations of dimension p≥1 that follow the Gaussian distribution with mean $\mu \in R^{p}$ and covariance matrix $\Sigma \in R^{p\times p}$, where without loss of generality, we assume μ=0. Let $\Theta :=\Sigma^{-1}$, and the empirical covariance matrix $S:=\frac{1}{n}\sum_{i=1}^{n}x_{i}^{T}x_{i}$. Given penalty parameter λ>0, the graphical lasso (GL) is the procedure to find the positive definite $\Theta \in R^{p\times p}$ such that:

(2) $$\underset{\Theta }{\mathrm{minimize}}\left\{-\log\det\Theta +\mathrm{trace}\left(S\Theta \right)+{\lambda ∥\Theta ∥}_{1}\;\right\},$$

where ${\left||\Theta |\right|}_{1}=\sum_{j\neq k}\left|\theta_{j,k}\right|.$ If we regard V:={1,⋯,p} as a vertex set, then we can construct an undirected graph with edge set $\left\{\left\{j,k\right\}|\theta_{j,k}\neq 0\right\}$, where set {j,k} denotes an undirected edge that connects the nodes j,k∈V.

If we take the subgradient of (2), then we find that the optimal solution $\Theta_{*}$ satisfies the condition:

(3) $$-\Theta_{*}^{-1}+S+\lambda \Phi ∋0,$$

where $\Phi =(\Phi_{j,k})$ is

$$\Phi_{j,k}=\left\{\begin{array}{cc} 1, & \theta_{j,k}^{*}>0\\ \left[-1,1\right], & \theta_{j,k}^{*}=0\\ -1, & \theta_{j,k}^{*}<0 \end{array}\right.\;.$$

### 2.2. ISTA for Graphical Lasso

In this subsection, we introduce the method for solving the GL problem (2) by the iterative shrinkage-thresholding algorithm (ISTA) proposed by [10], which is a proximal gradient method usually employed in dealing with nondifferentiable composite optimization problems.

Specifically, the general ISTA solves the following composite optimization problem:

(4) $$\underset{x}{\mathrm{minimize}\;}F\left(x\right):=f\left(x\right)+g\left(x\right),$$

where *f* and *g* are convex, with *f* differentiable and *g* possibly being nondifferentiable.

For the GL problem (2), we denote $f,g:R^{p\times p}\to R$ as

f(Θ):=−logdetΘ+trace(SΘ),

and

$$g\left(\Theta \right):={\lambda ∥\Theta ∥}_{1}.$$

If we define the quadratic approximation $Q_{\eta }:R^{p\times p}\times R^{p\times p}\to R$ w.r.t. f(Θ) and η>0 by

(5) $$\begin{array}{c} Q_{\eta }\left(\Theta^{\prime },\Theta \right):=f\left(\Theta \right)+\langle \Theta^{\prime }-\Theta ,\nabla f\left(\Theta \right)\rangle +\frac{1}{2\eta }\left|\right|\Theta^{\prime }-\Theta {\left|\right|}_{F}^{2}, \end{array}$$

then we can describe the ISTA as a procedure that iterates

(6) $$\begin{array}{cc} \Theta_{t+1} & =\arg\min_{\Theta }\left\{Q_{\eta_{t}}\left(\Theta ,\Theta_{t}\right)+g\left(\Theta \right)\right\} \end{array}$$

(7) $$\begin{array}{cc} \; & ={\mathrm{prox}}_{\eta_{t}g}\left(\Theta_{t}-\eta_{t}\nabla f\left(\Theta_{t}\right)\right), \end{array}$$

given initial value $\Theta_{0}$, where the value of step size $\eta_{t}>0$ may change at each iteration t=1,2,⋯, for efficient convergence purpose, and we use the proximal operator:

(8) $${\mathrm{prox}}_{g}\left(z\right):=\arg\min_{\theta }\{\frac{1}{2}{∥z-\theta ∥}_{2}^{2}+g\left(\theta \right)\}.$$

Note that the proximal operator of function $g={\lambda ||\Theta ||}_{1}$ is the soft-thresholding operator: the absolute value $|\theta_{i,j}|$ of each off-diagonal element $\theta_{i,j}$ with i≠j becoming either $\theta_{i,j}-\mathrm{sgn}\left(\theta_{i,j}\right)\lambda$ or zero (if $|\theta_{i,j}|<\lambda$). We use the following function for the operator in Section 3:

(9) $${\left[S_{\lambda }\left(\Theta \right)\right]}_{i,j}=\mathrm{sgn}\left(\theta_{i,j}\right)\left(\right|\theta_{i,j}{\left|-\lambda \right)}_{+}$$

where ${\left(x\right)}_{+}:=\max\left(x,0\right)$.

**Definition** **1.** *A differentiable function $f:R^{n\times p}\to R$ is said to have a Lipschitz-continuous gradient if there exists L>0 (Lipschitz constant) such that*

(10) $$||\nabla f\left(X\right)-{\nabla f\left(Y\right)||}_{F}\leq L||X-{Y||}_{F},\;\forall X,Y\in R^{n\times p}.$$

It is known that if we choose $\eta_{t}=\frac{1}{L}$ for each step in the ISTA that minimizes F(·), then the convergence rate is, at most, as follows [25]:

(11) $$F\left(\Theta_{t}\right)-F\left(\Theta_{*}\right)=O\left(\frac{1}{t}\right)$$

However, for the GL problem (2), we know neither the exact value of the Lipschitz constant *L* nor any nontrivial upper bound. [10] implement a backtracking line search option in Step 1 of Algorithm 1 below to handle this issue.

The backtracking line search enables us to compute the $\eta_{t}$ value for each time t=1,2,… by repeatedly multiplying $\eta_{t}$ by a constant c∈(0,1) until $\Theta_{t+1}≻0$ (Θ is positive definite) and

(12) $$f\left(\Theta_{t+1}\right)\leq Q_{\eta_{t}}\left(\Theta_{t+1},\Theta_{t}\right),$$

for the $\Theta_{t+1}$ in (7). Additionally, (12) is a sufficient condition for (11), which was derived in [25] (see the relationship between Lemma 2.3 and Theorem 3.1 in [25]).

The whole procedure is given in Algorithm 1.
**Algorithm 1** G-ISTA for problem (2).**Input**: S, tolerance ϵ>0, backtracking constant 0<c<1, initial value $\eta_{0}$, $\Theta_{0}$, t=0. **While**$t<t_{\max}$ (until convergence) **do**
    1:Backtracking line search: Continue to multiply $\eta_{t}$ by c until
$$\Theta_{t+1}≻0\;\mathrm{and}\;f\left(\Theta_{t+1}\right)\leq Q_{\eta_{t}}\left(\Theta_{t+1},\Theta_{t}\right)$$
for $\Theta_{t+1}:={prox}_{\eta_{t}g}\left(\Theta_{t}-\eta_{t}\nabla f\left(\Theta_{t}\right)\right)$.    2:Update iterate: $\Theta_{t+1}\leftarrow {prox}_{\eta_{t}g}\left(\Theta_{t}-\eta_{t}\nabla f\left(\Theta_{t}\right)\right)$.    3:Set next initial step size $\eta_{t+1}$ by the Barzilai—Borwein method.    4:t←t+1**end** **Output**: ϵ-optimal solution to problem (2), $\Theta_{*}=\Theta_{t+1}$.


### 2.3. Composite Self-Concordant Minimization

The notion of the self-concordant function was proposed in [26,27,28]. In the following, we say a convex function *f* is self-concordant with parameter M≥0 if

$$|f^{\prime \prime \prime }\left(x\right)|\leq Mf^{\prime \prime }{\left(x\right)}^{3/2},\;\mathrm{for}\;\mathrm{all}\;x\in \mathrm{dom}\;f.$$

where domf is the domain of *f*.

Reference [24] considered a composite version of self-concordant function minimization and provided a way to efficiently calculate the step size for the proximal gradient method for the GL problem without relying on the Lipschitz gradient assumption in (10). They proved that

f(Θ):=−logdetΘ+trace(SΘ)

in (2) is self-concordant and considers the following minimization:

$$F^{*}:=\underset{x}{\mathrm{minimize}\;}\left\{F\left(x\right):=f\left(x\right)+g\left(x\right)\right\},$$

where *f* is convex, differentiable, and self-concordant, and *g* is convex and possibly nondifferentiable. As for Algorithm 1, without using the backtracking line search, we can compute direction $d_{t}$ with initial step size $\eta_{t}$ as follows:

(13) $$d_{t}:={prox}_{\eta_{t}g}\left(\Theta_{t}-\eta_{t}\nabla f\left(\Theta_{t}\right)\right)-\Theta_{t},$$

where the operator prox is defined by (8). Then, we use the modified step size $\alpha_{t}$ to update $\Theta_{t+1}:=\Theta_{t}+\alpha_{t}d_{t}$, which can be determined by the direction $d_{t}$. After defining two parameters related to the direction: $\beta_{t}:=\eta_{t}^{-1}\left|\right|d_{t}{\left|\right|}_{F}^{2}$ and $\lambda_{t}:={\left(\langle \nabla^{2}f\left(\Theta_{t}\right)d_{t},d_{t}\rangle \right)}^{1/2}$, the modified step size can be obtained by

(14) $$\alpha_{t}:=\frac{\beta_{t}}{\lambda_{t}\left(\lambda_{t}+\beta_{t}\right)}\;.$$

By Lemma 12 in [24], if the modified step size $\alpha_{t}\in \left(0,1\right]$, then it can ensure a decrease in the objective function and guarantee convergence in the proximal gradient scheme. From (14), if $\lambda_{t}\geq 1$, then the condition $\alpha_{t}\in \left(0,1\right]$ is satisfied. Therefore, we only need to check the case when $\lambda_{t}<1$. If the condition $\alpha_{t}\in \left(0,1\right]$ does not hold, we can change the value of the initial $\eta_{t}$ (such as the bisection method) to influence the value of $d_{t}$ in (13) until the condition is satisfied.

### 2.4. Joint Graphical Lasso

Let N≥1,p≥1,K≥2, and $\left(x_{1},y_{1}\right),\ldots ,\left(x_{N},y_{N}\right)\in R^{p}\times \left\{1,\ldots ,K\right\}$, where each $x_{i}$ is a row vector. Let $n_{k}$ be the number of occurrences in $y_{1},\ldots ,y_{N}$ such that $y_{i}=k$, so that $\sum_{k=1}^{K}n_{k}=N$.

For each k=1,…,K, we define the empirical covariance matrix $S^{\left(k\right)}\in R^{p\times p}$ of the data $x_{i}$ as follows:

$$S^{\left(k\right)}:=\frac{1}{n_{k}}\sum_{i:y_{i}=k}x_{i}^{T}x_{i}.$$

Given the penalty parameters $\lambda_{1}>0$ and $\lambda_{2}>0$, the joint graphical lasso (JGL) is the procedure to find the positive definite matrix $\Theta^{\left(k\right)}\in R^{p\times p}$ for k=1,…,K, such that:

(15) $$\underset{\Theta }{\mathrm{minimize}}\left\{-\sum_{k=1}^{K}n_{k}\left\{\log\det\Theta^{\left(k\right)}-\mathrm{trace}\left(S^{\left(k\right)}\Theta^{\left(k\right)}\right)\right\}+\lambda_{1}\sum_{k=1}^{K}\sum_{i\neq j}\left|\theta_{k,i,j}\right|+P\left(\Theta \right)\right\},$$

where P(Θ) penalizes $\Theta :={\left[\Theta^{\left(1\right)},\ldots ,\Theta^{\left(K\right)}\right]}^{T}$. For example, ref. [13] suggested the following fused and group lasso penalties:

$$P_{F}\left(\Theta \right):=\lambda_{2}\sum_{k<l}\sum_{i,j}\left|\theta_{k,i,j}-\theta_{l,i,j}\right|$$

and

$$P_{G}\left(\Theta \right):=\lambda_{2}\sum_{i\neq j}{\left\{\sum_{k=1}^{K}\theta_{k,i,j}^{2}\right\}}^{1/2}\;,$$

where $\theta_{k,i,j}$ is the (i,j)-th element of $\Theta^{\left(k\right)}\in R^{p\times p}$ for k=1,⋯,K.

Unfortunately, there is no equation like (3) for the JGL to find the optimum $\Theta_{*}$. [13] considered the ADMM to solve the JGL problem. However, ADMM is quite time consuming for large-scale problems.

## 3. The Proposed Methods

In this section, we propose two efficient algorithms for solving the JGL problem. One is an extended ISTA based on the G-ISTA in Section 2.2, and the other is based on the step-size selection strategy introduced in Section 2.3.

### 3.1. ISTA for the JGL Problem

To describe the JGL problem, we define $f,g:R^{K\times p\times p}\to R$ by

(16) $$\begin{array}{cc} f(\Theta ) & :=-\sum_{k=1}^{K}n_{k}\left\{\log\det\Theta^{\left(k\right)}-\mathrm{trace}\left(S^{\left(k\right)}\Theta^{\left(k\right)}\right)\right\}, \end{array}$$

(17) $$\begin{array}{cc} g(\Theta ) & :=\lambda_{1}\sum_{k=1}^{K}\sum_{i\neq j}\left|\theta_{k,i,j}\right|+P\left(\Theta \right). \end{array}$$

Then, the problem (15) reduces to:

$$\underset{\Theta }{\mathrm{minimize}\;}F\left(\Theta \right):=f\left(\Theta \right)+g\left(\Theta \right)\;,$$

where the function *f* is convex and differentiable, and *g* is convex and nondifferentiable. Therefore, the ISTA is available for solving the JGL problem (15).

The main difference between the G-ISTA and the proposed method is that the latter needs to simultaneously consider *K* categories of graphical models in the JGL problem (15). What is more, there are two combined penalties in g(Θ), which complicate the proximal operator in the ISTA procedure. Consequently, the operator for the proposed method is not a simple soft thresholding operator, as it is for the G-ISTA method.

If we define the quadratic approximation $Q_{\eta_{t}}:R^{K\times p\times p}\to R$ of $f(\Theta_{t})$ by:

$$\begin{array}{c} Q_{\eta_{t}}\left(\Theta ,\Theta_{t}\right):=f\left(\Theta_{t}\right)+\sum_{k=1}^{K}\langle \Theta^{\left(k\right)}-\Theta_{t}^{\left(k\right)},\nabla f\left(\Theta_{t}^{\left(k\right)}\right)\rangle +\frac{1}{2\eta_{t}}\sum_{k=1}^{K}\left|\right|\Theta^{\left(k\right)}-\Theta_{t}^{\left(k\right)}{\left|\right|}_{F}^{2}, \end{array}$$

then the update iteration is simplified as:

$$\begin{array}{cc} \Theta_{t+1} & =\underset{\Theta }{\mathrm{argmin}\;}\left\{Q_{\eta_{t}}\left(\Theta ,\Theta_{t}\right)+g\left(\Theta \right)\right\}\\ \; & ={\mathrm{prox}}_{\eta_{t}g}\left(\Theta_{t}-\eta_{t}\nabla f\left(\Theta_{t}\right)\right). \end{array}$$

Nevertheless, the Lipschitz gradient constant of f(Θ) is unknown over the whole domain in the JGL problem. Therefore, our approach needs a backtracking line search to calculate step size $\eta_{t}$. We show the details in Algorithm 2.
**Algorithm 2** ISTA for problem (15).**Input**: S, tolerance ϵ>0, backtracking constant 0<c<1, initial step size $\eta_{0}$, initial iterate $\Theta_{0}$. **For**t=0,1,⋯, (until convergence) **do**
    1:Backtracking line search: Continue to multiply $\eta_{t}$ by c until
$$\begin{array}{c} f\left(\Theta_{t+1}\right)\leq Q_{\eta_{t}}\left(\Theta_{t+1},\Theta_{t}\right)\;\mathrm{and}\;\Theta_{t+1}^{\left(k\right)}≻0\;\mathrm{for}\;k=1,\cdots ,K. \end{array}$$
for $\Theta_{t+1}:={\mathrm{prox}}_{\eta_{t}g}\left(\Theta_{t}-\eta_{t}\nabla f\left(\Theta_{t}\right)\right)$.    2:Update iterate: $\Theta_{t+1}\leftarrow {\mathrm{prox}}_{\eta_{t}g}\left(\Theta_{t}-\eta_{t}\nabla f\left(\Theta_{t}\right)\right)$.    3:Set next initial step size $\eta_{t+1}$. See details in Section 3.3.**end** **Output**: optimal solution to problem (15), $\Theta_{*}=\Theta_{t+1}$.


In the update of $\Theta_{t+1}$, we need to compute the proximal operators for the fused and group lasso penalties. In the following, for each of them, the problem can be divided into the fused lasso problems [29] and group lasso problems [30,31] for $\theta_{i,j}\in R^{K}$, i,j=1,…,p. We apply the solutions given by (20) and (21) below.

#### 3.1.1. Fused Lasso Penalty $P_{F}$

By the definition of the proximal operator in the update step, we have:

(18) $$\begin{array}{cc} \Theta_{t+1}=\arg\min_{\Theta }\left\{\frac{1}{2}\sum_{k=1}^{K}\left|\right|\Theta^{\left(k\right)}-\Theta_{t}^{\left(k\right)}+\eta_{t}\nabla f\left(\Theta_{t}^{\left(k\right)}\right){\left|\right|}_{F}^{2}\right. & +\eta_{t}\lambda_{1}\sum_{k=1}^{K}\sum_{i\neq j}\left|\theta_{k,i,j}\right|\\ \; & \left.+\eta_{t}\lambda_{2}\sum_{k<l}\sum_{i,j}\left|\theta_{k,i,j}-\theta_{l,i,j}\right|\right\}. \end{array}$$

Problem (18) is separable with respect to the elements $\theta_{k,i,j}$ in $\Theta^{\left(k\right)}\in R^{p\times p}$; hence, the proximal operator can be computed in a componentwise manner: Let $A=\Theta_{t}-\eta_{t}\nabla f\left(\Theta_{t}\right)$; then, problem (18) reduces to the following for i=1,⋯,p, j=1,⋯,p:

(19) $$\begin{array}{c} \underset{\theta_{1,i,j},\cdots ,\theta_{K,i,j}}{\mathrm{argmin}\;}\left\{\frac{1}{2}\sum_{k=1}^{K}{\left(\theta_{k,i,j}-a_{k,i,j}\right)}^{2}+\eta_{t}\lambda_{1}1_{i\neq j}\sum_{k=1}^{K}|\theta_{k,i,j}|+\eta_{t}\lambda_{2}\sum_{k<l}|\theta_{k,i,j}-\theta_{l,i,j}\left|\right\}\right\}, \end{array}$$

where $1_{i\neq j}$ is an indicator function, the value of which is 1 only when i≠j.

The problem (19) is known as the fused lasso problem [29,32] given $a_{k,i,j}$ for k=1,⋯,K. In particular, let $\alpha :=\eta_{t}\lambda_{1}1_{i\neq j}$ and $\beta :=\eta_{t}\lambda_{2}$. When i≠j, α≠0 and β>0, the solution to (19) can be obtained through the soft thresholding operator based on the solution when α=0 by the following Lemma.

**Lemma** **1.** *(*[33]*) Denote the solution to parameters α and β as $\theta_{i}\left(\alpha ,\beta \right)$, and then the solution $\theta_{i}\left(\alpha ,\beta \right)$ of the fused lasso problem:*

(20) $$\frac{1}{2}\sum_{i=1}^{n}{\left(y_{i}-\theta_{i}\right)}^{2}+\alpha \sum_{i=1}^{n}|\theta_{i}|+\beta \sum_{i=1}^{n-1}\left|\theta_{i}-\theta_{i+1}\right|$$

*is given by ${\left[S_{\alpha }\left(\theta \left(0,\beta \right)\right)\right]}_{i}$ when $y_{1},\cdots ,y_{n}\in R$ are given for n≥1.*

Additionally, rather efficient algorithms are available for solving the fused lasso problem (20) when α=0 (i.e., θ(0,β) ) such as [32,34,35].

#### 3.1.2. Group Lasso Penalty $P_{G}$

By definition, the update of $\Theta_{t+1}$ for the group lasso penalty $P_{G}\left(\Theta \right)$ is as follows:

$$\begin{array}{cc} \Theta_{t+1}=\arg\min_{\Theta }\left\{\frac{1}{2}\sum_{k=1}^{K}\left|\right|\Theta^{\left(k\right)}-\Theta_{t}^{\left(k\right)}+\eta_{t}\nabla f\left(\Theta_{t}^{\left(k\right)}\right){\left|\right|}_{F}^{2}\right. & +\eta_{t}\lambda_{1}\sum_{k=1}^{K}\sum_{i\neq j}\left|\theta_{k,i,j}\right|\\ \; & \left.+\eta_{t}\lambda_{2}\sum_{i\neq j}{\left(\sum_{k=1}^{K}\theta_{k,i,j}^{2}\right)}^{1/2}\right\}. \end{array}$$

Similarly, let $A=\Theta_{t}-\eta_{t}\nabla f\left(\Theta_{t}\right)$; then, the problem becomes the following for i=1,⋯,p, j=1,⋯,p:

$$\begin{array}{c} \underset{\theta_{1,i,j},\cdots ,\theta_{K,i,j}}{\mathrm{argmin}\;}\left\{\frac{1}{2}\sum_{k=1}^{K}{\left(\theta_{k,i,j}-a_{k,i,j}\right)}^{2}+\eta_{t}\lambda_{1}1_{i\neq j}\sum_{k=1}^{K}\left|\theta_{k,i,j}\right|+\eta_{t}\lambda_{2}1_{i\neq j}{\left(\sum_{k=1}^{K}\theta_{k,i,j}^{2}\right)}^{1/2}\right\}. \end{array}$$

We have $\theta_{k,i,j}=a_{k,i,j}$ for i=j. In addition, for i≠j, the solution [31,36,37] is given by

(21) $$\theta_{k,i,j}=S_{\eta_{t}\lambda_{1}}\left(a_{k,i,j}\right){\left(1-\frac{\eta_{t}\lambda_{2}}{\sqrt{\sum_{k=1}^{K}S_{\eta_{t}\lambda_{1}}{\left(a_{k,i,j}\right)}^{2}}}\right)}_{+}.$$

### 3.2. Modified ISTA for JGL

Thus far, we have seen that f(Θ) in the JGL problem (15) is not globally Lipschitz gradient continuous. The ISTA may not be efficient enough for the JGL case because it includes the backtracking line search procedure for this case, which needs to evaluate the objective function and the positive definiteness of $\Theta_{t+1}$ in Step 1 of Algorithm 2 and is inefficient when the evaluation is expensive.

In this section, we modify Algorithm 2 to Algorithm 3 based on the step-size selection strategy in Section 2.3, which takes advantage of the properties of the self-concordant function. The self-concordant function does not rely on the Lipschitz gradient assumption on the function f(Θ) [24], and we can eliminate the need for the backtracking line search.

**Lemma** **2.** *(*[38]*) Self-concordance is preserved by scaling and addition: if f is a self-concordant function and a constant a≤1, then af is self-concordant. If $f_{1},f_{2}$ are self-concordant, then $f_{1}+f_{2}$ is self-concordant.*

By Lemma 2, the function f(Θ) in (16) is self-concordant. In Algorithm 3, for the initial step size of $\eta_{t}$ in each iteration, we use the Barzilai–Borwein method [39]. We apply the step-size mechanism in Section 2.3, which is employed in Steps 3–5 of Algorithm 3.
**Algorithm 3** Modified ISTA (M-ISTA).**Input**: S, tolerance ϵ>0, initial step size $\eta_{0}$, initial iterate $\Theta_{0}$. **For**t=0,1,⋯, (until convergence) **do**
    1:Initialize $\eta_{t}$.    2:Compute
$$\begin{array}{c} d_{t}:={\mathrm{prox}}_{\eta_{t}g}\left(\Theta_{t}-\eta_{t}\nabla f\left(\Theta_{t}\right)\right)-\Theta_{t}. \end{array}$$    3:Compute
$$\begin{array}{c} \beta_{t}:=\eta_{t}^{-1}\left|\right|d_{t}{\left|\right|}_{F}^{2} \end{array}$$
and
$$\begin{array}{c} \lambda_{t}:=\sum_{k=1}^{K}\sqrt{n_{k}}\left|\right|{\left(\Theta_{t}^{\left(k\right)}\right)}^{-1}d_{t}^{\left(k\right)}{\left|\right|}_{F}. \end{array}$$    4:Determine the step size $\alpha_{t}:=\frac{\beta_{t}}{\lambda_{t}\left(\lambda_{t}+\beta_{t}\right)}$.    5:If $\alpha_{t}>1$, then set $\eta_{t}:=\eta_{t}/2$ and go back to Step 2.    6:Update $\Theta_{t+1}:=\Theta_{t}+\alpha_{t}d_{t}$.**end** **Output**: optimal solution to problem (15), $\Theta_{*}=\Theta_{t+1}$.


There is no backtracking procedure in this algorithm that guarantees the positive definiteness of $\Theta_{t+1}$, as in Step 1 of Algorithm 2. We next show how to ensure the positive definiteness of $\Theta_{t+1}$ in the iterations of Algorithm 3.

**Lemma** **3.** *(*[40]*, Theorem 2.1.1) Let f be a self-concordant function, and let x∈domf. Additionally, if*

$$W\left(x\right)=\left\{y|{\left(\langle \nabla^{2}f\left(x\right)\left(y-x\right),y-x\rangle \right)}^{1/2}\leq 1\right\},$$

*then W(x)⊂domf.*

In Algorithm 3, because we know $\alpha_{t}:=\frac{\beta_{t}}{\lambda_{t}\left(\lambda_{t}+\beta_{t}\right)}<1$ with $\beta_{t}>0$ and $\lambda_{t}>0$ by Steps 3–5. Thus, we have $\alpha_{t}\lambda_{t}<1$:

$$\alpha_{t}\lambda_{t}:=\alpha_{t}{\left(\langle \nabla^{2}f\left(\Theta_{t}\right)d_{t},d_{t}\rangle \right)}^{1/2}<1,$$

which implies,

$${\left(\langle \nabla^{2}f\left(\Theta_{t}\right)\left(\Theta_{t+1}-\Theta_{t}\right),\Theta_{t+1}-\Theta_{t}\rangle \right)}^{1/2}<1.$$

Hence, from Lemma 3, we see that $\Theta_{t+1}$ stays in the domain and maintains positive definiteness.

### 3.3. Theoretical Analysis

For multiple Gaussian graphical models, Honorio and Samaras [14] and Hara and Washio [17] provided lower and upper bounds for the optimal solution $\Theta_{*}$. However, the models they considered are different than the JGL. To the best of our knowledge, no related research has provided the bounds of the optimal solution $\Theta_{*}$ for the JGL problem (15).

In the following section, we show the bounds of the optimal solution $\Theta_{*}$ for the JGL and the iterates $\Theta_{t}$ generated by Algorithms 2 and 3, which are applied to both fused and group lasso-type penalties.

**Proposition** **1.** The optimal solution $\Theta_{*}$ of the problem (15) satisfies

$$\max_{1\leq k\leq K}\frac{n_{k}}{p\lambda_{c}+n_{k}\left|\right|S^{\left(k\right)}{\left|\right|}_{2}}\leq \left|\right|\Theta_{*}^{\left(k\right)}{\left|\right|}_{2}\leq \frac{Np}{\lambda_{1}}+\sum_{k=1}^{K}\sum_{i=1}^{p}{\left(s_{k,i,i}\right)}^{-1},$$

*where $\lambda_{c}:=\sqrt{K\lambda_{1}^{2}+2K\lambda_{1}\lambda_{2}+\lambda_{2}^{2}}$, and $s_{k,i,i}$ is the i-th diagonal element of $S^{\left(k\right)}$.*

For the proof, see Appendix A.1.

Note that the objective function value F(Θ) always decreases with the increase in iteration in both algorithms due to [25] (Remark 3.1) and Lemma 12 in [24]. Therefore, the following inequality holds for Algorithms 2 and 3:

(22) $$F\left(\Theta_{t+1}\right)\leq F\left(\Theta_{t}\right)\;\mathrm{for}\;t=0,1,\cdots .$$

Then, based on the condition (22), we provide the explicit bounds of iterates ${\left\{\Theta_{t}\right\}}_{t=0,1\cdots }$ in Algorithms 2 and 3 for the JGL problem (15).

**Proposition** **2.** *Sequence ${\left\{\Theta_{t}\right\}}_{t=0,1,\cdots ,}$ generated by Algorithms 2 and 3 can be bounded:*

$$m\leq \left|\right|\Theta_{t}{\left|\right|}_{2}\leq M,$$

*where $M:=\left|\right|\Theta_{0}{\left|\right|}_{F}+\frac{2Np}{\lambda_{1}}+2\sum_{k=1}^{K}\sum_{i=1}^{p}{s_{k,i,i}}^{-1}$, $m:=e^{-\frac{C_{1}}{n_{m}}}M^{\left(1-Kp\right)}$, $n_{m}=\max_{k}n_{k}$, and constant $C_{1}:=F\left(\Theta_{0}\right)$.*

For the proof, see Appendix A.2.

With the help of Propositions 1 and 2, and the following Lemma, we can obtain the range of the step size that ensures the linear convergence rate of Algorithm 2.

**Lemma** **4.** *Let $\Theta_{t}$ be t-th iterate in Algorithm 2. Denote $\lambda_{min}$ and $\lambda_{max}$ as the minimum and maximum eigenvalues of the corresponding matrix, respectively. Define*

$$a_{k}:=\min\left\{\lambda_{\min}\left(\Theta_{t}^{\left(k\right)}\right),\lambda_{\min}\left(\Theta_{*}^{\left(k\right)}\right)\right\},\;b_{k}:=\max\left\{\lambda_{\max}\left(\Theta_{t}^{\left(k\right)}\right),\lambda_{\max}\left(\Theta_{*}^{\left(k\right)}\right)\right\}$$

*and $n_{l}=\min_{k=1\cdots ,K}n_{k}$, $n_{m}=\max_{k=1\cdots ,K}n_{k}$, $a_{l}=\min_{k=1\cdots ,K}a^{\left(k\right)}$, and $b_{m}=\max_{k=1\cdots ,K}b^{\left(k\right)}$. The sequence ${\left\{\Theta_{t}\right\}}_{t=0,1,\cdots }$ generated by Algorithm 2 satisfies*

$$\left|\right|\Theta_{t+1}-\Theta_{*}{\left|\right|}_{F}\leq \gamma_{t}\left|\right|\Theta_{t}-\Theta_{*}{\left|\right|}_{F}$$

*with the convergence rate $\gamma_{t}:=\max\left\{\frac{\eta_{t}n_{m}}{a_{l}^{2}}-1,1-\frac{\eta_{t}n_{l}}{b_{m}^{2}}\right\}$.*

**Proof.**  It can be easily extended by Lemma 3 in [10]. □

Lemma 4 implies that to obtain the convergence rate $\gamma_{t}<1$, we require:

(23) $$0<\eta_{t}<\frac{2a_{l}^{2}}{n_{m}}\;.$$

After using Propositions 1 and 2, we can obtain the bounds of $a_{l}$. Further, we can obtain the step size $\eta_{t}$ that satisfies (23) and guarantee s the linear convergence rate $\left(\gamma_{t}<1\right)$. However, the step size is quite conservative in practice. Hence, we consider the Barzilai–Borwein method for implementation and regard the step size $\eta_{t}$ that satisfies (23) as a safe choice. When the number of backtracking iterations in Step 1 of Algorithm 2 exceeds the given maximum number to fulfill the backtracking line search condition, we can use the safe step size $\eta_{t}$ for the subsequent calculations. In Section 4.2.3, we confirm the linear convergence rate of the proposed ISTA by experiment.

## 4. Experiments

In this section, we evaluate the performance of the proposed methods on both synthetic and real datasets, and we compare the following algorithms:

- ADMM: the general ADMM method proposed by [13].
- FMGL: the proximal Newton-type method proposed by [23].
- ISTA: the proposed method in Algorithm 2.
- M-ISTA: the proposed method in Algorithm 3.

We perform all the tests in R Studio on a Macbook Air with 1.6 GHz Intel Core i5 and 8 GB memory. The wall times are recorded as the run times for the four algorithms.

### 4.1. Stopping Criteria and Model Selection

In the experiments, we consider two stopping criteria for the algorithms.

1. Relative error stopping criterion:

$$\frac{\sum_{k=1}^{K}\left|\right|\Theta_{t+1}^{\left(k\right)}-\Theta_{t}^{\left(k\right)}{\left|\right|}_{F}}{\max\{\sum_{k=1}^{K}||\Theta_{t}^{\left(k\right)}|{|}_{F},1\}}\leq \epsilon .$$

2. Objective error stopping criterion:

$$F\left(\Theta_{t}\right)-F\left(\Theta_{*}\right)\leq \epsilon .$$

ϵ is a given accuracy tolerance; we terminate the algorithm if the above error is smaller than ϵ or the maximum number of iterations exceeds 1000. We use the objective error for convergence rate analysis and the relative error for the time comparison.

The JGL model is affected by regularized parameters $\lambda_{1}$ and $\lambda_{2}$. For selecting the parameters, we use the *V*-fold crossvalidation method. First, the dataset is randomly split into *V* segments of equal size, a single subset (test data), estimated by the other V−1 subsets (training data), is evaluated, and the subset is changed for the test to repeat *V* times so that each subset is used.

Let $S_{v}^{\left(k\right)}$ be the sample covariance matrix of the *v*-th ( v=1,…,V) segment for class k=1,⋯,K. We estimate the inverse covariance matrix by the remaining V−1 subsets ${\hat{\Theta }}_{\lambda ,-v}^{\left(k\right)}$ and choose $\lambda_{1}$ and $\lambda_{2}$, which minimize the average predictive negative log-likelihood as follows:

$$CV\left(\lambda_{1},\lambda_{2}\right)=\sum_{v=1}^{V}\sum_{k=1}^{K}\left\{n_{k}\mathrm{trace}\left(S_{v}^{\left(k\right)}{\hat{\Theta }}_{\lambda ,-v}^{\left(k\right)}\right)-\mathrm{logdet}{\hat{\Theta }}_{\lambda ,-v}^{\left(k\right)}\right\}$$

### 4.2. Synthetic Data

The performance of the proposed methods was assessed on synthetic data in terms of the number of iterations, the execution time, the squared error, and the receiver operating characteristic (ROC) curve. We follow the data generation mechanism described in [41] with some modifications for the JGL model. We put the details in Appendix B.

#### 4.2.1. Time Comparison Experiments

**Figure 1:**
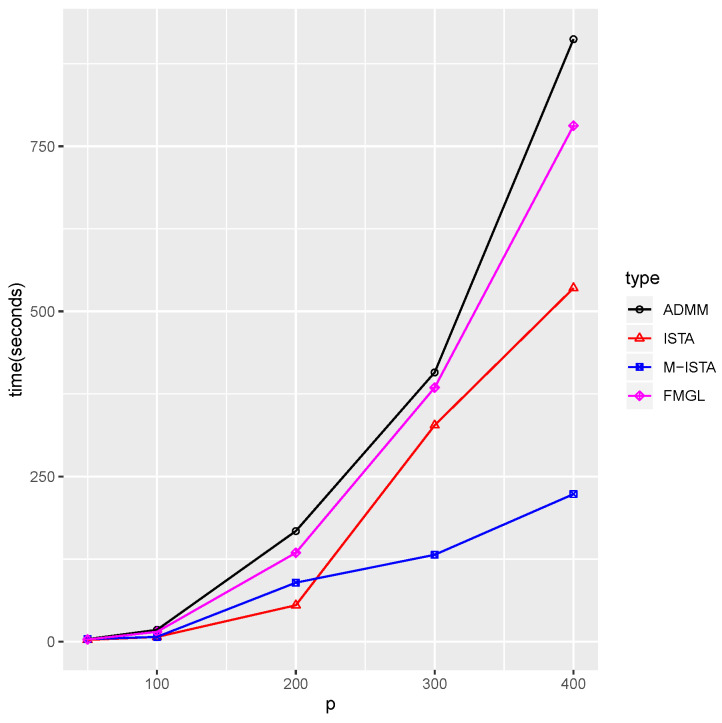
Plot of time comparison under different *p*. Setting $\lambda_{1}=0.1$, $\lambda_{2}=0.05$, K=2, and N=200.

We vary $p,N,K\;\mathrm{and}\;\lambda_{1}$ to compare the execution time of our proposed methods with that of the existing methods. We consider only the fused penalty in our proposed method for a fair comparison in the experiments because the FMGL algorithm applies only to the fused penalty. First, we compare the performance among different algorithms under various dimensions *p*, which are shown in Figure 1.

Figure 1 shows that the execution time of the FMGL and ADMM increases rapidly as *p* increases. In particular, we observe that the M-ISTA significantly outperforms when *p* exceeds 200. The ISTA shows better performance than the three methods when *p* is less than 200, but it requires more time as *p* grows, compared to the M-ISTA. It is reasonable to consider that evaluating the objective function in the backtracking line search at every iteration increases the computational burden, especially when *p* increases, which means that the M-ISTA is a good choice for these cases. Furthermore, the ISTA can be a good candidate when the evaluation is inexpensive.

Table 2 summarizes the performance of the four algorithms under different parameter settings to achieve a given precision, ϵ, of the relative error. The results presented in Table 2 reveal that when we increase the number of classes *K*, all the algorithms require more time than usual. Moreover, the execution time of ADMM becomes huge among them. When we vary $\lambda_{1}$, the algorithms become more efficient as the value increases. For most instances, the M-ISTA and ISTA outperform the existing methods, such as ADMM and FMGL. For the exceptional cases ($p=20,k=2,N=60,\lambda_{1}=0.1$ and $\lambda_{2}=0.05$), the M-ISTA and ISTA are still comparable with the FMGL and faster than ADMM.

#### 4.2.2. Algorithm Assessment

We generate the simulation data as described in Appendix B and regard the synthetic inverse covariance matrices $\Theta^{\left(k\right)}$ as the true values for our assessment experiments.

First, we assessed our proposed method by drawing an ROC curve, which displays the number of true positive edges (i.e., TP edges) selected compared to the number of false positive edges (i.e., FP edges) selected. We say that an edge (i,j) in the *k*-th class is selected in estimate ${\hat{\Theta }}^{\left(k\right)}$ if element ${\hat{\theta }}_{k,i,j}\neq 0$, and the edges are true positive edges selected if the precision matrix element $\theta_{k,i,j}\neq 0$ and false positive edges selected if the precision matrix element $\theta_{k,i,j}=0$, where the two quantities are defined by

$$TP=\sum_{k=1}^{K}\sum_{i,j}1\left(\theta_{k,i,j}\neq 0\right)\cdot 1\left({\hat{\theta }}_{k,i,j}\neq 0\right)\;$$

and

$$FP=\sum_{k=1}^{K}\sum_{i,j}1\left(\theta_{k,i,j}=0\right)\cdot 1\left({\hat{\theta }}_{k,i,j}\neq 0\right)\;,$$

where 1(·) is the indicator function.

To confirm the validity of the proposed methods, we compare the ROC figures of the fused penalty and group penalty. We fix the parameters $\lambda_{2}$ for each curve and change the $\lambda_{1}$ value to obtain various numbers of selected edges because the sparsity penalty parameter $\lambda_{1}$ can control the number of selected total edges.

**Figure 2:**
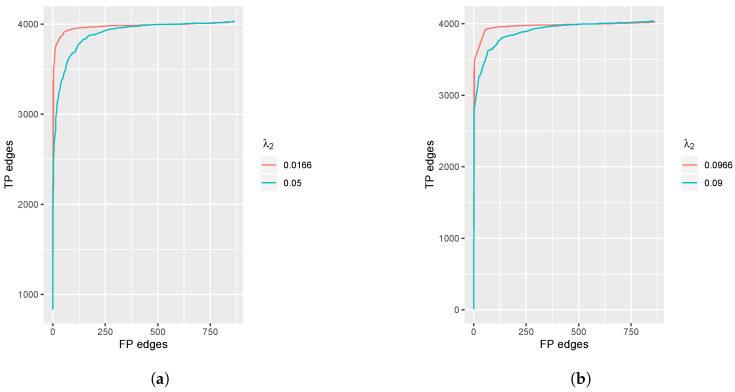
Plot of true positive edges vs. false positive edges selected. Setting p=50, K=2. (**a**) The fused penalty; (**b**) The group penalty.

We show the ROC curves for fused and group lasso penalties in Figure 2a,b respectively. From the figures, we observe that both penalties show highly accurate predictions for the edge selections. The result of $\lambda_{2}=0.0166$ in the fused penalty case is better than that in $\lambda_{2}=0.05$. Additionally, the result of $\lambda_{2}=0.0966$ in the group penalty case is better than that in $\lambda_{2}=0.09$, which means that if we select the tuning parameters properly, then we can obtain precise results while simultaneously meeting our different model demands.

**Figure 3:**
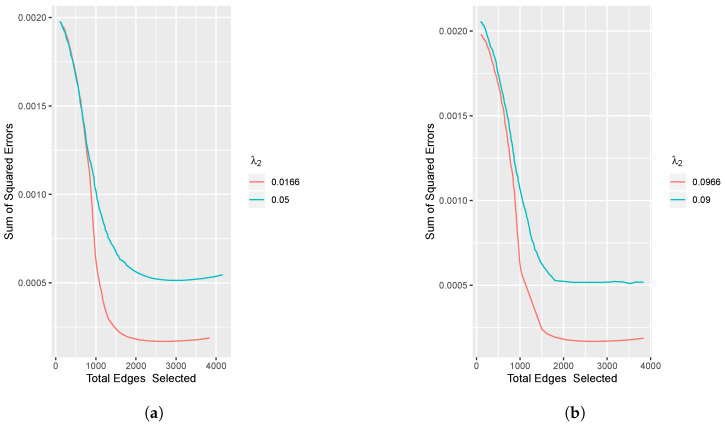
Plot of the mean squared errors vs. total edges selected. Setting p=50, K=2. (**a**) The fused penalty; (**b**) The group penalty.

Then, Figure 3a,b display the mean squared error (MSE) between the estimated values and true values.

$$MSE=\frac{2}{Kp(p-1)}\sum_{k=1}^{K}\sum_{i<j}{\left({\hat{\theta }}_{k,i,j}-\theta_{k,i,j}\right)}^{2},$$

where ${\hat{\theta }}_{k,i,j}$ is the value estimated by the proposed method, and $\theta_{k,i,j}$ is the true precision matrix value we used in the data generation.

The figures illustrate that when the total number of edges selected increases, the errors decrease and finally achieve relatively low values.

Overall, the proposed method shows competitive efficiency not only in computational time but also in accuracy.

#### 4.2.3. Convergence Rate

**Figure 4:**
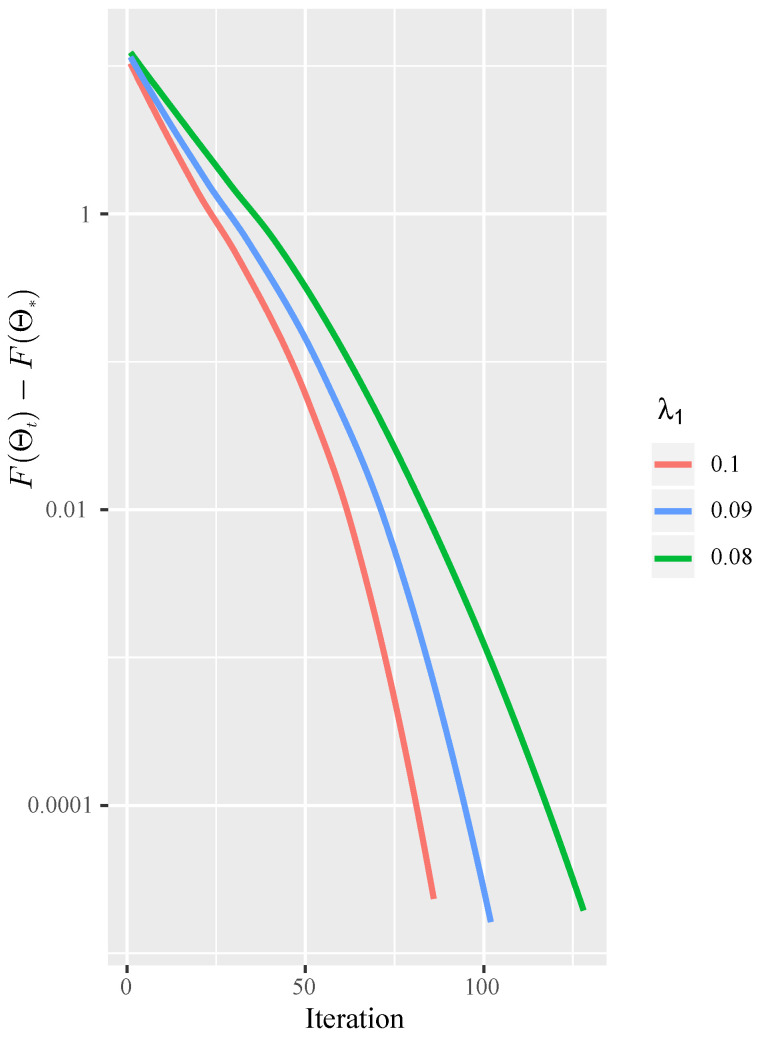
Plot of $\log(F\left(\Theta_{t}\right)-F\left(\Theta_{*}\right))$ vs. the number of iterations with different $\lambda_{1}$ values. Setting p=200,N=400,K=2 and $\lambda_{2}=0.05$.

This section shows the convergence rate of the ISTA for solving the JGL problem (15) in practice, with $\lambda_{1}=0.1$, 0.09 and 0.08. We recorded the number of iterations to achieve the different tolerance of $F\left(\Theta_{t}\right)-F\left(\Theta_{*}\right)$ in Figure 4 and ran it on a synthetic dataset, with p=200, K=2, $\lambda_{2}=0.05$, and N=400. The figure reveals that as $\lambda_{1}$ decreases, more iterations are needed to converge to the specified tolerance. Moreover, the figure shows the linear convergence rate of the proposed ISTA method, which corroborate the theoretical analysis in Section 3.3.

### 4.3. Real Data

In this section, we use two different real datasets to demonstrate the performance of our proposed method and visualize the result.

Firstly, we used the presidential speeches dataset in [42] for the experiment to jointly estimate common links across graphs and show the common structure. The dataset contains 75 most-used words (features) from several big speeches of the 44 US presidents (samples). In addition, we used the clustering result in [42], where the authors split the 44 samples into two groups with similar features, and then we obtained two classes of samples (K=2).

**Figure 5:**
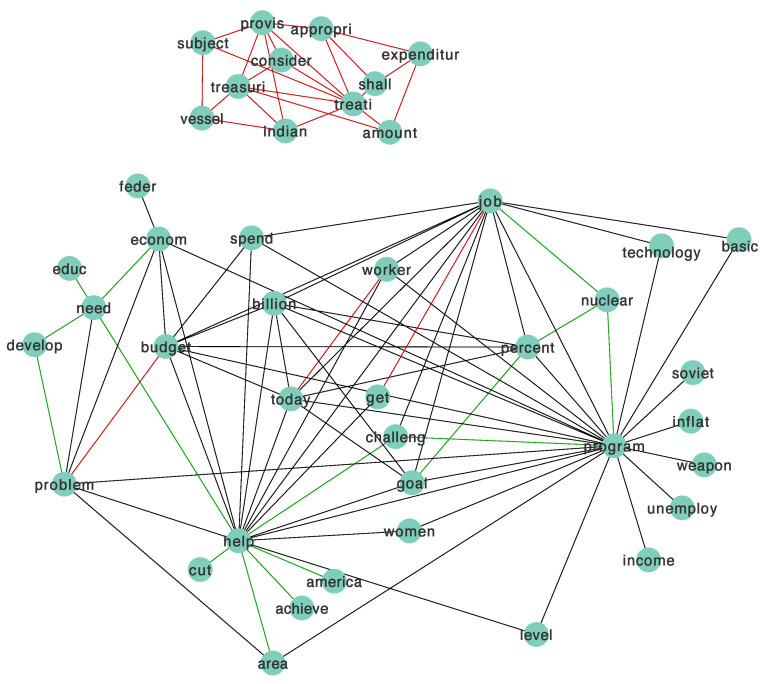
Network figure of the words in president speeches dataset.

We used Cytoscape [43] to visualize the results when $\lambda_{1}=1.9$ and $\lambda_{2}=0.16$. We chose these relatively large tuning parameters for better interpretation of the network figure. Figure 5 shows the relationship network graph of the high-frequency words identified by the JGL model with the proposed method. As shown in the figure, each node represents a word, and the edges demonstrate the relationships between words.

We use different colors to show various structures. The black edges are a common structure between the two classes, the red edges are the specific structures for the first class (k=1), and the green edges are for the second class (k=2). Figure 5 shows a subnetwork on the top with red edges, meaning there are relationships among those words, and the connections only exist in the first group.

We compared the time cost among four algorithms and show the results in Table 3. We used the crossvalidation method (V=6) described in Section 4.1 to select the optimal tuning parameters ($\lambda_{1}=0.1$, $\lambda_{2}=0.05$). In addition, we manually chose the other two pairs of parameters for more comparisons.

Table 3 shows that ISTA outperforms the other three algorithms, and our proposed methods offer stable performance when varying the parameters, while ADMM is the slowest in most cases.

Secondly, we use a breast cancer dataset [44] for time comparison. There are 250 samples and 1000 genes in the dataset, with 192 control samples and 58 case samples (K=2). Furthermore, we extract 200 genes with the highest variances among the original genes. The tuning parameter pair $(\lambda_{1}=0.01$, $\lambda_{2}=0.0166)$ was chosen by the crossvalidation method. Table 3 exhibits that our proposed methods (ISTA and M-ISTA) outperform ADMM and FMGL, and M-ISTA shows the best performance in the breast cancer dataset.

## 5. Discussion

We propose two efficient proximal gradient descent procedures with and without the backtracking line search option for the joint graphical lasso. The first (Algorithm 2) does not require extra variables, unlike ADMM, which needs manual tuning the Lagrangian penalty parameters ρ in [13] and storing and calculating dual variables. Moreover, we reduce the update iterate step to subproblems that can be solved efficiently and precisely by lasso-type problems. Based on Algorithm 2, we modified the step-size selection by extending the strategy in [24] to the second one (Algorithm 3), which does not rely on the Lipschitz assumption. Additionally, the second does not require a backtracking line search, significantly reducing the computation time needed to evaluate objective functions.

From the theoretical perspective, we reach the linear convergence rate for the ISTA. Furthermore, we derive the lower and upper bounds of the solution to the JGL problem and the iterates in the algorithms, guaranteeing that the ISTA converges linearly. Numerically, the methods are demonstrated on simulated and real datasets to illustrate their robust and efficient performance over state-of-the-art algorithms.

For further computational improvement, the most expensive step in the algorithms is to calculate the inversion of matrices required by the gradient of f(Θ). Both algorithms have a complexity of $O(Kp^{3})$ per iteration. Moreover, we can solve the matrix inversion problem with more efficient algorithms with lower complexity. In addition, we can also use the faster computation procedure in [13] to decompose the optimization problem for the proposed methods and regard it as preprocessing. Overall, the proposed methods are highly efficient for the joint graphical lasso problem.

## Figures and Tables

**Table 1:** Efficient JGL procedures.

Model	ADMM	Proximal Newton	Proximal Gradient
GL [4]	[8]	[12]	[10]
JGL [13]	[13]	[23]	Current Paper
		(for fused penalty)	(for fused and group penalties)

**Table 2:** Computational time under different settings.

Parameters Setting						Computational Time			
*p*	*K*	*N*	$\lambda_{1}$	$\lambda_{2}$	**precision** ϵ	ADMM	FMGL	ISTA	M-ISTA
20	2	60	0.1	0.05	0.00001	10.506 s	**1.158 s**	2.174 s	1.742 s
	3					1.879 min	4.267 s	**3.357 s**	3.668 s
	5		1	0.5		1.123 min	10.556 s	4.216 s	**2.874 s**
30	2	120	0.1	0.05	0.0001	10.095 s	5.259 s	**2.690 s**	4.857 s
	3					2.014 min	38.562 s	**14.722 s**	31.870 s
	5		1	0.5		2.447 min	15.819 s	22.431 s	**12.113 s**
50	2	600	0.02	0.005	0.0001	6.427 s	10.228 s	7.213 s	**4.625 s**
			0.03			6.240 s	8.925 s	6.645 s	**4.023 s**
			0.04			7.025 s	9.381 s	6.144 s	**3.993 s**
200	2	400	0.09	0.05	0.0001	4.050 min	1.874 min	2.289 min	**35.038 s**
			0.1			4.569 min	1.137 min	1.340 min	**24.852 s**
			0.12			3.848 min	1.881 min	1.443 min	**18.367 s**

**Table 3:** Time comparison result of two real datasets.

Dataset	Parameters Setting			Computational Time			
	$\lambda_{1}$	$\lambda_{2}$	Precision ϵ	ADMM	FMGL	ISTA	M-ISTA
Speeches	0.1	0.05	0.0001	19.969 s	4.977 min	**11.829 s**	12.867 s
	0.2	0.1		4.661 min	3.209 min	**11.560 s**	12.682 s
	0.5	0.25		5.669 min	1.490 min	**11.043 s**	12.788 s
Breast cancer	0.1	0.0166	0.0001	3.809 min	7.937 min	1.305 min	**1.158 min**
	0.2	0.02		6.031 min	5.198 min	1.503 min	**1.230 min**
	0.3	0.03		5.499 min	2.265 min	1.188 min	**1.061 min**

## Data Availability

Publicly available datasets were analyzed in this paper. Presidential speeches dataset: https://www.presidency.ucsb.edu, accessed on 5 November 2021; Breast cancer dataset: https://www.rdocumentation.org/packages/doBy/versions/4.5-15/topics/breastcancer, accessed on 5 November 2021.

## Abbreviations

The following abbreviations are used in this manuscript:

ADMM	alternating direction method of multipliers
FMGL	fused multiple graphical lasso algorithm
FP	false positive
G-ISTA	graphical iterative shrinkage-thresholding algorithm
GL	graphical lasso
ISTA	iterative shrinkage-thresholding algorithm
JGL	joint graphical lasso
M-ISTA	modified iterative shrinkage-thresholding algorithm
MSE	mean squared error
ROC	receiver operating characteristic
TP	true positive

## Appendix A. Proofs of Propositions

### Appendix A.1. Proof of Proposition 1

We first introduce the Lagrange dual problem of (15). By introducing the auxiliary variables $Z=\{Z^{\left(1\right)},\cdots ,Z^{\left(K\right)}\}$, we can rewrite the problem as follows:

$$\begin{array}{c} \min_{\Theta }f\left(\Theta \right)+g\left(\Theta \right)\\ \mathrm{subject}\;\mathrm{to}\;Z=\Theta \end{array}$$

Then, the Lagrange function of the above is given by:

$$L\left(\Theta ,Z,\Lambda \right)=f\left(\Theta \right)+g\left(Z\right)+\sum_{k=1}^{K}\langle \Lambda^{\left(k\right)},\Theta^{\left(k\right)}-Z^{\left(k\right)}\rangle ,$$

where $\Lambda =\left\{\Lambda^{\left(1\right)},\cdots ,\Lambda^{\left(K\right)}\right\},\Lambda^{\left(k\right)}\in R^{p\times p}$ are dual variables. To obtain the dual problem, we minimize the primal variables as follows:

$$\begin{array}{cc} \min_{\Lambda ,Z}L\left(\Theta ,Z,\Lambda \right) & =\min_{\Theta }\left\{f\left(\Theta \right)+\sum_{k=1}^{K}\langle \Lambda^{\left(k\right)},\Theta^{\left(k\right)}\rangle \right\}-\max_{Z}\left\{-g\left(Z\right)-\sum_{k=1}^{K}\langle \Lambda^{\left(k\right)},-Z^{\left(k\right)}\rangle \right\}\\ \; & =\min_{\Theta }\left\{f\left(\Theta \right)+\sum_{k=1}^{K}\langle \Lambda^{\left(k\right)},\Theta^{\left(k\right)}\rangle \right\}-g^{*}\left(\Lambda \right)\\ \; & =\min_{\Theta }\left\{\sum_{k=1}^{K}\langle \Lambda^{\left(k\right)}+n_{k}S^{\left(k\right)},\Theta^{\left(k\right)}\rangle -\sum_{k=1}^{K}n_{k}\mathrm{logdet}\Theta^{\left(k\right)}\right\}-g^{*}\left(\Lambda \right). \end{array}$$

Taking derivative of the function:

$$L_{1}:=\sum_{k=1}^{K}\langle \Lambda^{\left(k\right)}+n_{k}S^{\left(k\right)},\Theta^{\left(k\right)}\rangle -\sum_{k=1}^{K}n_{k}\mathrm{logdet}\Theta^{\left(k\right)},$$

$$\nabla_{\Theta^{\left(k\right)}}L_{1}=0,$$

We obtain

(A1) $$n_{k}S^{\left(k\right)}+\Lambda^{\left(k\right)}=n_{k}{\left(\Theta^{\left(k\right)}\right)}^{-1}$$

for k=1,⋯,K. Substitute the Equation (A1) into the dual problem $\min_{\Theta ,Z}L\left(\Theta ,Z,\Lambda \right)$, then it becomes:

$$\begin{array}{cc} \min_{\Theta ,Z} & L\left(\Theta ,Z,\Lambda \right)=\sum_{k=1}^{K}n_{k}p+\sum_{k=1}^{K}n_{k}\mathrm{logdet}\left(S^{\left(k\right)}+\frac{1}{n_{k}}\Lambda^{\left(k\right)}\right)-g^{*}\left(\Lambda \right). \end{array}$$

Hence, we can obtain the duality gap [38] (the primal problem minus the dual problem) as follows:

$$\begin{array}{cc} f(\Theta )+g(Z) & -\sum_{k=1}^{K}n_{k}p-\sum_{k=1}^{K}n_{k}\left\{\mathrm{logdet}\Theta^{\left(k\right)}\right\}+g^{*}\left(\Lambda \right)\\ \; & =\sum_{k=1}^{K}n_{k}\mathrm{trace}\left(S^{\left(k\right)}\Theta^{\left(k\right)}\right)+g\left(Z\right)-\sum_{k=1}^{K}n_{k}p+g^{*}\left(\Lambda \right), \end{array}$$

when the gap value is 0, the optimal solution is found. Because the conjugate function $g^{*}\left(\Lambda \right)$ is the indicator function, the value is hence 0 for the optimal solution.

Firstly, for the group penalty $P_{G}\left(\Theta \right)$, the duality gap is

(A2) $$\begin{array}{cc} \sum_{k=1}^{K}\left[n_{k}\mathrm{trace}\left(S^{\left(k\right)}\Theta_{*}^{\left(k\right)}\right)-n_{k}p\right] & +\lambda_{1}\sum_{k=1}^{K}\sum_{i\neq j}\left|{\theta_{*}}_{k,i,j}\right|+\lambda_{2}\sum_{i\neq j}\sqrt{\sum_{k=1}^{K}{{\theta_{*}}_{k,i,j}}^{2}}=0. \end{array}$$

From Equation (A2), we obtain

$$\begin{array}{cc} \lambda_{1}\left|\right|\Theta_{*}{\left|\right|}_{1} & =-\sum_{k=1}^{K}n_{k}\mathrm{trace}\left(S^{\left(k\right)}\Theta_{*}^{\left(k\right)}\right)-\lambda_{2}\sum_{i\neq j}\sqrt{\sum_{k=1}^{K}{{\theta_{*}}_{k,i,j}}^{2}}+\sum_{k=1}^{K}n_{k}p+\sum_{k=1}^{K}\sum_{i=1}^{p}\lambda_{1}\left|{\theta_{*}}_{k,i,i}\right|\\ \; & \leq \sum_{k=1}^{K}n_{k}p+\sum_{k=1}^{K}\sum_{i=1}^{p}\lambda_{1}\left|{\theta_{*}}_{k,i,i}\right|. \end{array}$$

From Equation (A1), we have the following relationship of diagonal elements,

$$\begin{array}{c} {\theta_{k,i,i}}^{*}=diag{\left(S^{\left(k\right)}+\frac{1}{n_{k}}\Lambda_{*}^{\left(k\right)}\right)}^{-1}, \end{array}$$

and due to dual variable $\Lambda_{k,i,i}^{*}>0$, for k=1,⋯,K. Hence,

$$\begin{array}{cc} \left|\right|\Theta_{*}{\left|\right|}_{1} & \leq \frac{1}{\lambda_{1}}\sum_{k=1}^{K}n_{k}p+\sum_{k=1}^{K}\sum_{i=1}^{p}diag{\left(S^{\left(k\right)}+\frac{1}{n_{k}}\Lambda_{*}^{\left(k\right)}\right)}^{-1}\\ \; & \leq \frac{1}{\lambda_{1}}\sum_{k=1}^{K}n_{k}p+\sum_{k=1}^{K}\sum_{i=1}^{p}diag{\left(S^{\left(k\right)}\right)}^{-1}. \end{array}$$

By $\left|\right|\Theta_{*}{\left|\right|}_{2}\leq \left|\right|\Theta_{*}{\left|\right|}_{F}\leq \left|\right|\Theta_{*}{\left|\right|}_{1}$, we obtain the upper bound:

(A3) $$\begin{array}{c} \left|\right|\Theta_{*}{\left|\right|}_{2}\leq \left|\right|\Theta_{*}{\left|\right|}_{F}\leq \frac{1}{\lambda_{1}}\sum_{k=1}^{K}n_{k}p+\sum_{k=1}^{K}\sum_{i=1}^{p}{s_{k,i,i}}^{-1}. \end{array}$$

The proof is similar for the fused penalty $P_{F}\left(\Theta \right)$; therefore, we omit it here. Next, we continue to prove the lower bound of $\Theta_{*}$.

Firstly, for the group penalty $P_{G}\left(\Theta \right)$. Let $E^{\left(k\right)}$ be non-negative p×p matrix satisfying $-E_{k,i,j}\leq \theta_{k,i,j}\leq E_{k,i,j}$. Introducing the Lagrange multipliers $\Gamma^{\left(k\right)}$ and $\Gamma_{0}^{\left(k\right)}$ for k=1,⋯,K. This procedure is similar to the way in [17].

Then, the new Lagrange problem becomes,

$$\begin{array}{cc} \max_{\Theta ,E}\min_{\Gamma ,\Gamma_{0}} & \left\{f\left(\Theta \right)-\sum_{k=1}^{K}\sum_{i\neq j}\lambda_{1}E_{k,i,j}-\lambda_{2}\sum_{i\neq j}\sqrt{\sum_{k=1}^{K}E_{k,i,j}^{2}}\right.\\ \; & \left.-\sum_{k=1}^{K}tr\left(\Gamma^{\left(k\right)}\Theta^{\left(k\right)}\right)-tr\left(abs\left(\Gamma^{\left(k\right)}\right)E^{\left(k\right)}\right)-tr\left(\Gamma_{0}^{\left(k\right)}E^{\left(k\right)}\right)\right\}, \end{array}$$

Taking derivative w.r.t $\Theta^{\left(k\right)}$ and $E_{k,i,j}$, we obtain the following equations:

(A4) $$n_{k}{\Theta^{\left(k\right)}}^{-1}-n_{k}S^{\left(k\right)}-\Gamma^{\left(k\right)}=0,$$

(A5) $$\begin{array}{cc} -\lambda_{1}-\lambda_{2}\frac{E_{k,i,j}}{\sqrt{\sum_{k=1}^{K}E_{k,i,j}^{2}}}+\left|\Gamma_{k,i,j}\right|+\Gamma_{0}^{\left(k\right)} & =0,\;\mathrm{for}\;i\neq j, \end{array}$$

(A6) $$\begin{array}{cc} |\Gamma_{k,i,j}|+\Gamma_{0}^{\left(k\right)} & =0,\;\mathrm{for}\;i=j. \end{array}$$

When i≠j, from Equation (A5),

$$\begin{array}{cc} |\Gamma_{k,i,j}| & \leq \lambda_{1}+\lambda_{2}\frac{E_{k,i,j}}{\sqrt{\sum_{k=1}^{K}E_{k,i,j}^{2}}}\\ |\Gamma_{k,i,j}{|}^{2} & \leq {\left(\lambda_{1}+\lambda_{2}\frac{E_{k,i,j}}{\sqrt{\sum_{k=1}^{K}E_{k,i,j}^{2}}}\right)}^{2}\\ \; & =\lambda_{1}^{2}+2\lambda_{1}\lambda_{2}\frac{E_{k,i,j}}{\sqrt{\sum_{k=1}^{K}E_{k,i,j}^{2}}}+\lambda_{2}^{2}\frac{E_{k,i,j}^{2}}{\sum_{k=1}^{K}E_{k,i,j}^{2}}\\ \; & \leq \lambda_{1}^{2}+2\lambda_{1}\lambda_{2}+\lambda_{2}^{2}\frac{E_{k,i,j}^{2}}{\sum_{k=1}^{K}E_{k,i,j}^{2}}. \end{array}$$

Taking summation of each k,

$$\sum_{k=1}^{K}{\left|\Gamma_{k,i,j}\right|}^{2}\leq K\lambda_{1}^{2}+2K\lambda_{1}\lambda_{2}+\lambda_{2}^{2}.$$

Then,

(A7) $$\sqrt{\sum_{k=1}^{K}{\left|\Gamma_{k,i,j}\right|}^{2}}\leq \sqrt{K\lambda_{1}^{2}+2K\lambda_{1}\lambda_{2}+\lambda_{2}^{2}}.$$

From (A4) and (A7), we have the following relationship

$$\begin{array}{cc} \left|\right|{\Theta^{\left(k\right)}}^{-1}\left|\right|\leq \left|\right|\frac{1}{n_{k}}\Gamma^{\left(k\right)}+S^{\left(k\right)}{\left|\right|}_{2} & \leq \frac{1}{n_{k}}\left|\right|\Gamma^{\left(k\right)}{\left|\right|}_{2}+\left|\right|S^{\left(k\right)}{\left|\right|}_{2}\\ \; & \leq \frac{p}{n_{k}}\max_{i,j}|\Gamma_{k,i,j}\left|+|\right|S^{\left(k\right)}{\left|\right|}_{2}\\ \; & \leq \frac{p}{n_{k}}\max_{k}\max_{i,j}|\Gamma_{k,i,j}\left|+|\right|S^{\left(k\right)}{\left|\right|}_{2}\\ \; & \leq \frac{p(\sqrt{K\lambda_{1}^{2}+2K\lambda_{1}\lambda_{2}+\lambda_{2}^{2}})}{n_{k}}+\left|\right|S^{\left(k\right)}{\left|\right|}_{2}. \end{array}$$

The last equation holds because

$$\max_{k}\max_{i,j}\left|\Gamma_{k,i,j}\right|\leq \sqrt{\sum_{k=1}^{K}{\left|\Gamma_{k,i,j}\right|}^{2}}.$$

We only consider the case when i≠j for $max_{i,j}\left|\Gamma_{ij}^{\left(k\right)}\right|$, because from Equations (A5) and (A6), we know $|\Gamma_{ij}^{\left(k\right)}\left|>\right|\Gamma_{ii}^{\left(k\right)}|$. Overall, the lower bound is

$$\frac{n_{k}}{p\sqrt{K\lambda_{1}^{2}+2K\lambda_{1}\lambda_{2}+\lambda_{2}^{2}}+n_{k}\left|\right|S^{\left(k\right)}{\left|\right|}_{2}}.$$

The lower bound of fused penalty can be derived in similar way.

### Appendix A.2. Proof of Proposition 2

By Equation (22) and convexity of F(Θ), it is easy to obtain

$$\left|\right|\Theta_{t}-\Theta_{*}{\left|\right|}_{F}\leq \left|\right|\Theta_{0}-\Theta_{*}{\left|\right|}_{F}.$$

Since $\left|\right|\cdot {\left|\right|}_{2}\leq \left|\right|\cdot {\left|\right|}_{F}$, then

$$\begin{array}{cc} \left|\right|\Theta_{t}{\left|\right|}_{2}-\left|\right|\Theta_{*}{\left|\right|}_{2} & \leq \left|\right|\Theta_{t}-\Theta_{*}{\left|\right|}_{2}\\ \; & \leq \left|\right|\Theta_{t}-\Theta_{*}{\left|\right|}_{F}\\ \; & \leq \left|\right|\Theta_{0}-\Theta_{*}{\left|\right|}_{F}. \end{array}$$

Hence,

$$\begin{array}{cc} \left|\right|\Theta_{t}{\left|\right|}_{2} & \leq \left|\right|\Theta_{0}-\Theta_{*}{\left|\right|}_{F}+\left|\right|\Theta_{*}{\left|\right|}_{2}\\ \; & \leq \left|\right|\Theta_{0}{\left|\right|}_{F}+2||\Theta_{*}{\left|\right|}_{F}. \end{array}$$

Then, by Equation (A3), we can complete the proof of the upper bound.

To prove the lower bound, denote

$$\begin{array}{cc} a_{t}^{\left(k\right)} & =\lambda_{\min}\left(\Theta_{t}^{\left(k\right)}\right)\\ {\left(a_{t}\right)}_{l} & =\min_{k=1,\cdots ,K}a_{t}^{\left(k\right)}. \end{array}$$

By the definition of the matrix norm, we have

$$\left|\right|\Theta_{t}^{\left(k\right)}{\left|\right|}_{2}\geq a_{t}^{\left(k\right)}\geq {\left(a_{t}\right)}_{l}.$$

Denote the upper bound of $\left|\right|\Theta_{t}{\left|\right|}_{2}$ as *M*, and that of $\left|\right|\Theta_{t}{\left|\right|}_{2}$ as $M^{\left(k\right)}$, for k=1,⋯,K. By definition of tensor norm, we have $M\geq \left|\right|\Theta_{t}{\left|\right|}_{2}\geq \left|\right|\Theta_{t}^{\left(k\right)}{\left|\right|}_{2}\geq {\left(a_{t}\right)}_{l}$.

Let constant $C_{1}:=f\left(\Theta_{0}\right)+g\left(\Theta_{0}\right)$. By the Equation (22), we have

$$C_{1}\geq f\left(\Theta_{t}\right)+g\left(\Theta_{t}\right).$$

Note that $S⪰0,\Theta_{t}≻0$ implies $tr(S\Theta_{t})\geq 0$ and because $g(\Theta_{t})\geq 0$

$$\begin{array}{cc} C_{1} & \geq -\sum_{k=1}^{K}n_{k}\mathrm{logdet}\Theta_{t}^{\left(k\right)}\\ \; & =-\sum_{k=1}^{K}n_{k}\log\left(\Pi_{i=1}^{p}\lambda_{i}\right). \end{array}$$

Let the eigenvalues of $\Theta_{t}^{\left(k\right)}$ as $\lambda_{1}\leq \lambda_{2}\leq \cdots \leq \lambda_{p}$. Then $a_{t}^{\left(k\right)}=\lambda_{1}\leq \lambda_{p}\leq M^{\left(k\right)}$, hence,

$$\Pi_{i=1}^{p}\left(\lambda_{i}\right)=a_{t}^{\left(k\right)}\cdot \lambda_{2}\cdot \cdots \lambda_{p}\leq a_{t}^{\left(k\right)}\cdot {M^{\left(k\right)}}^{\left(p-1\right)}.$$

Then,

$$\sum_{k=1}^{K}n_{k}\log\left(\Pi_{i=1}^{p}\lambda_{i}\right)\leq \sum_{k=1}^{K}n_{k}\left[loga_{t}^{\left(k\right)}+\left(p-1\right)logM^{\left(k\right)}\right].$$

Let the coefficient $n_{k}$ of the term which contains ${\left(a_{t}\right)}_{l}$ in $-\sum_{k=1}^{K}n_{k}loga_{t}^{\left(k\right)}$ as $n_{x}$, then

$$\sum_{k=1}^{K}n_{k}loga_{t}^{\left(k\right)}=n_{x}log{\left(a_{t}\right)}_{l}+\sum_{k\neq x}n_{k}loga_{t}^{\left(k\right)}.$$

Because

$$M^{\left(k\right)}\leq M,$$

denote $n_{m}=\max_{i=1\cdots ,K}n_{k}$, then,

$$\begin{array}{c} \sum_{k=1}^{K}n_{k}loga_{t}^{\left(k\right)}\leq n_{m}log{\left(a_{t}\right)}_{l}+n_{m}\left(K-1\right)logM. \end{array}$$

Hence,

$$\begin{array}{cc} C_{1} & \geq -\sum_{k=1}^{K}n_{k}\log\left(\Pi_{i=1}^{p}\left(\lambda_{i}\right)\right)\\ \; & \geq -\sum_{k=1}^{K}n_{k}\left[loga_{t}^{\left(k\right)}+\left(p-1\right)logM^{\left(k\right)}\right]\\ \; & \geq -n_{m}log{\left(a_{t}\right)}_{l}-n_{m}\left(K-1\right)log\;M\\ \; & \;-Kn_{m}\left(p-1\right)logM. \end{array}$$

Then, we can obtain

$$\begin{array}{cc} log{\left(a_{t}\right)}_{l} & \geq -K\left(p-1\right)logM-\left(K-1\right)logM-\frac{C_{1}}{n_{m}}\\ {\left(a_{t}\right)}_{l} & \geq e^{\left(1-Kp\right)logM-\frac{C_{1}}{n_{m}}}. \end{array}$$

Hence, the lower bound is proved:

$$\left|\right|\Theta_{t}{\left|\right|}_{2}\geq \left|\right|\Theta_{t}^{\left(k\right)}{\left|\right|}_{2}\geq {\left(a_{t}\right)}_{l}\geq e^{-\frac{C_{1}}{n_{m}}}M^{\left(1-Kp\right)}.$$

## Appendix B. Data Generation

We generate $n_{k}$ samples independently and identically distributed observations from a multivariate normal distribution $N\{0,{\left({\hat{\Theta }}^{\left(k\right)}\right)}^{-1}\}$, where $\Theta^{\left(k\right)}$ is the inverse covariance matrix of the *k*-th category. Specifically, we generate *p* points randomly on a unit space and calculate their pairwise distances. Then, we find the *m*-nearest neighbors point by this distance. We connect any two points that are *m*-nearest neighbors of each other. The integer *m* determines for the degree of sparsity of the data, and *m* values range from 4 to 9 in our experiments.

Additionally, we add heterogeneity to the common structure by building extra individual connections in the following way: we randomly choose a pair of symmetric zero elements, $\theta_{k,i,j}=\theta_{k,j,i}=0$, and replace them with a value uniformly generated from the [−1,−0,5]∪[0.5,1] interval. This operation is repeated M/2 times, where *M* is the number of off-diagonal nonzero elements in $\Theta^{\left(k\right)}$.
